# Modeling age‐dependent developmental changes in the expression of genes involved in citrulline synthesis using pig enteroids

**DOI:** 10.14814/phy2.14565

**Published:** 2020-11-12

**Authors:** Mahmoud A. Mohammad, Inka C. Didelija, Barbara Stoll, Douglas G. Burrin, Juan C. Marini

**Affiliations:** ^1^ USDA/ARS Children’s Nutrition Research Center Baylor College of Medicine Houston TX USA; ^2^ Pediatric Critical Care Medicine Department of Pediatrics Baylor College of Medicine Houston TX USA; ^3^ Food Science and Nutrition Department National Research Centre Dokki, Giza Egypt

## Abstract

**Background:**

Age‐dependent changes in the intestinal gene expression of enzymes involved in the metabolism of citrulline and arginine are well characterized. Enteroids, a novel ex‐vivo model that recreates the three‐dimensional structure of the intestinal crypt‐villus unit, have shown to replicate molecular and physiological profiles of the intestinal segment from where they originated (“location memory”).

**Objective:**

The present study tested the hypothesis that enteroids recapitulate the developmental changes observed in vivo regarding citrulline production in pigs (“developmental memory”).

**Methods:**

Preterm (10‐ and 5‐d preterm) and term pigs at birth, together with 7‐ and 35‐d‐old pigs were studied. Gene expression was measured in jejunal samples and in enteroids derived from this segment. Whole body citrulline production was measured by isotope dilution and enteroid citrulline production by accumulation in the media.

**Results:**

With the exception of arginase I and inducible nitric oxide synthase, all the genes investigated expressed in jejunum were expressed by enteroids. In the jejunum, established markers of development (lactase and sucrase‐isomaltase), as well as genes that code for enzymes involved in the production and utilization of citrulline and arginine, underwent the ontogenic changes described in the literature. However, enteroid expression of these genes, as well as citrulline production, failed to recapitulate the changes observed in vivo.

**Conclusions:**

Under culture conditions used in our study, enteroids derived from jejunal crypts of pigs at different ages failed to replicate the gene expression observed in whole tissue and whole body citrulline production. Additional extracellular cues may be needed to reproduce the age‐dependent phenotype.

## Graphical abstract

Enteroids, a novel ex‐vivo model that recreates the 3D structure of the intestinal crypt‐villus unit, have shown to replicate molecular and physiological profiles of the intestinal segment from where they originated (‘location memory’). Age‐dependent changes in the intestinal gene expression of enzymes involved in the metabolism of citrulline and arginine are well characterized. In this study, we showed that enteroids do not recapitulate the developmental changes observed in vivo and thus have no ‘developmental memory’.

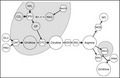

## 1 INTRODUCTION

In the gastrointestinal tract, profound ontogenic changes take place from late fetal life through the neonatal period, to weaning. The interaction of gene expression and endogenous regulatory mechanisms, as well as environmental influences (including microbial colonization and establishment of the microbiota), result in phenotypic changes in the epithelial cells that line the small intestine (Lebenthal & Lebenthal, 1999). Among these changes, the ability of epithelial cells to produce citrulline, the endogenous precursor for arginine synthesis, is crucial for the production of arginine which is an indispensable amino acid during the neonatal period (Wu, Jaeger, Bazer, & Rhoads, 2004). Low plasma citrulline concentration has been associated with pulmonary hypertension in human neonates (Pearson et al., 2001) and reduced citrulline synthesis has been shown to precede the onset of necrotizing enterocolitis (NEC) in premature pigs (Robinson et al., 2018). Because citrulline and arginine supplementation have been shown to rescue hypoxia‐induced pulmonary hypertension in newborn pigs (Ananthakrishnan et al., 2009; Fike et al., 2015) and to prevent NEC in premature infants (Amin et al., 2002; Shah, Shah, & Kelly, 2017) the adequate endogenous supply of citrulline by the small intestine for arginine synthesis may be crucial for physiological adaptations after birth.

**Figure 1:**
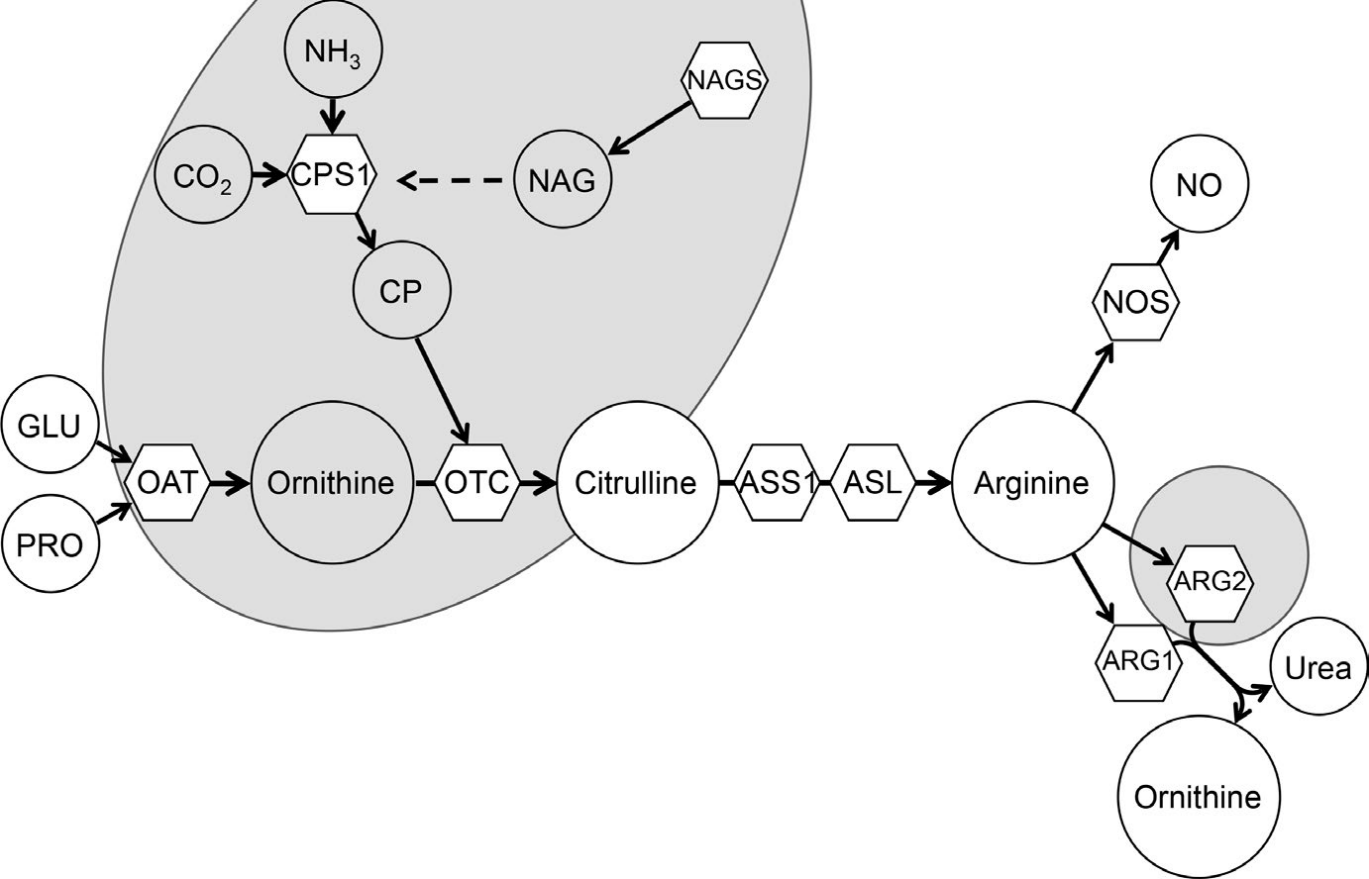
Pathway for the endogenous synthesis of arginine. Metabolites (circles) and enzymes (hexagons) are depicted. GLU, glutamate; PRO, proline; CO_2_, carbon dioxide; NH_3_, ammonia; CP, carbamoylphosphate; NAG, N‐acetylglutamate; NO, nitric oxide; OAT, ornithine aminotransferase; CPS1, carbamoylphosphate synthetase I; NAGS, N‐acetylglutamate synthase; OTC, ornithine transcarbamylase; ASS1, argininosuccinate synthase; ASL, argininosuccinate lyase; NOS, nitric oxide synthase; ARG1 and ARG2, arginase I and II, respectively. Enzymes within the grayed area are mitochondrial and the rest cytosolic. Whereas CPS1, NAGS, and OTC are only present in enterocytes (and hepatocytes), OAT, ASS1, and ASL are widely expressed in other tissues (mainly in kidney)

The intestinal expression of the enzymes involved in the synthesis of citrulline from ornithine (which occur only in enterocytes) and arginine metabolism undergo changes that have been well characterized in neonatal mice, rats, pigs, and humans (De Jonge, Dingemanse, De Boer, Lamers, & Moorman, 1998; Herzfeld & Raper, 1976; Hurwitz & Kretchmer, 1986; Kohler et al., 2008; Wu, Knabe, & Kim, 2004). The expression and activity of carbamyl phosphate synthase 1 (CPS1; Figure 1), ornithine transcarbamylase (OTC) and ornithine transaminase (OAT), enzymes responsible for the production of citrulline, seem to decline from birth. Enzymes that utilize citrulline for arginine synthesis (argininosuccinate synthase and lyase, ASS1 and ASL, respectively) are only present during the neonatal period and disappear in the adult. In contrast, arginase II (ARG2) expression increases with age.

Enteroids are a novel ex vivo model that contain intestinal stem cells and differentiated intestinal epithelial cells within a three‐dimensional (3D) structure containing crypt‐, villus‐, and lumen like domains similar to the in vivo architecture of the small intestinal epithelium (Moore et al., 2015). Furthermore, it has been shown that enteroids can maintain the molecular and physiological profiles of the intestinal segment from where they originated and thus have a “location memory” (Kozuka et al., 2017; Middendorp et al., 2014). This location‐specific function seems to be intrinsically programmed in the adult stem cells and their differentiation fate is independent of location‐specific extracellular signals. However, it is not clear if these stem cells have a similar “developmental memory”.

Due to its intestinal development and physiology, the pig is considered a more appropriate species to model human infant (patho)physiological processes than rodents. Not only do pigs and humans have more mature digestive functions shortly after birth than rodents (Downes, 2018; Neal‐Kluever, Fisher, Grylack, Kakiuchi‐Kiyota, & Halpern, 2019), but in mice the elongation of villi and crypts, and the establishment of a generative zone are not fully completed until after weaning (Hirano, 1986) whereas in pigs villi are fully formed 10 days before term (~90% gestation)(Robinson et al., 2018). Whereas premature pigs are susceptible to dietary induction of NEC (Sangild et al., 2006), recapitulating many of the key elements observed in humans, the rodent NEC models require additional conditions (including hypothermia, hypoxia, and endotoxemia) to simulate this disease (Premkumar et al., 2014). In addition, while mouse enteroids differentiate spontaneously and human enteroids require the removal of Wnt3a from the media (Khalil et al., 2016; Kozuka et al., 2017), pig enteroids may also benefit from Wnt3a removal. Because of all these common features between humans and pigs, the pig is an ideal model to study the development of enteroids and for their testing and validation vis‐a‐vis ontogenic changes that take place in the live animal. Therefore, the present study was conducted to test the hypothesis that enteroids recapitulate the developmental changes observed in vivo regarding citrulline production and the expression of enzymes involved in the metabolism of citrulline and arginine in preterm and term pigs at birth, and pigs during the suckling and post weaning period.

## 2 MATERIAL AND METHODS

### 2.1 General

Domestic conventionally reared crossbred pigs were obtained from a local commercial swine farm. All animal procedures were approved by the Baylor College of Medicine Institutional Animal Care and Use Committee.

### 2.2 Animals and tissue collection

Pigs of five different age groups (−10, −5, 0, 7, 35 d) were studied from a herd where natural vaginal birth at term normally occurs at ~ 115 days gestation. Ten‐day preterm (*n* = 8, 4 males, 4 female) and 5‐day preterm (*n* = 8, 5 males, 3 female) pigs were obtained after cesarean section at post‐conception day 105 (*n* = 4 sows) and 110 (*n* = 4 sows), respectively. Term pigs (*n* = 6, from 2 litters, 3 males, 3 female) were delivered naturally. An umbilical catheter was placed within 2–3 hr of birth for tracer delivery and blood sampling under light isoflurane anesthesia. Preterm and term pigs were euthanized within 4–6 hr of birth and had no access to any source of feed. One‐week‐old pigs (*n* = 8 from 2 litters, 5 males, 3 female) were littermates of the term pigs and stayed with the sow until the day of the study. Five‐week‐old pigs (*n* = 8, 4 males, 4 female) were weaned at the farm at 21 d of age and brought to the research facility at 28 d of age. During the 7 day acclimation period pigs were fed the same soybean meal/corn diet from weaning until the day of the study. After a 4–6 hr (7‐d‐oldpigs) or 10 hr (35‐d‐old pigs) feed restriction period, pigs were implanted with a carotid catheter under light isoflurane anesthesia for tracer delivery and blood sampling. A bolus dose of [^15^N] citrulline (50 µmol/kg) was given and multiple samples collected over 70 min. At the end of the sampling period pigs were euthanized. Proximal jejunum was collected immediately and, after flushing thoroughly with phosphate buffered saline (PBS), subsamples were frozen in liquid nitrogen or collected for crypt isolation.

### 2.3 Enteroid culture

Intestinal crypts were isolated from the proximal jejunum of the pigs. Crypt isolation and growth parameters were based on methods described by others (Sato et al., 2009). Briefly, 1–2 cm sections of jejunum were flushed and rinsed with PBS containing gentamicin and amphotericin B. After opening the intestinal sections and scraping the mucosa gently to remove villi, the jejunum was rinsed in cold PBS to remove cell debris. The tissue was then incubated in EDTA‐chelating buffer at 4°C for 30 min in a shaker. Crypts were then released from the jejunum by a second, more vigorous scraping and filtered using a 100‐µm filter (Falcon Cell Strainer; Corning, Durham NC). After centrifugation at 150 g for 10 min at 4°C, crypts were suspended in 5 ml PBS. The number of crypts were then assessed using a light microscope. Crypts were pelleted again by centrifugation and resuspended in 150–300 µl Matrigel (Corning Biosciences, Tewksbury, MA), and 3 30 µl drops/well were plated. Matrigel was solidified by incubating the 24‐well plate in a 37°C incubator (5% CO2) for 10 min. Complete media with growth factor (500 µl, CMGF+) were added to each well, and plates returned to the 37°C incubator. Culture medium was obtained from the Digestive Disease Center (Texas Medical Center, Houston, TX) and was composed of DMEM/12 containing key growth factors including Wnt3a conditioned media, Rspo1‐Fc‐conditioned media, Noggin conditioned media, human recombinant EGF, and human Gastrin I. Enteroid cultures were monitored daily, media were changed every other day and enteroids were frozen in Cell Culture Freezing Medium (Gibco Recovery Medium, ThermoFisher) usually 7 d after plating (Passage 0).

To generate the experimental enteroids, Passage 0 enteroids were coded and randomized in order to maintain the operator blinded to the treatment. Enteroids were then thawed and passed two times (Passage 2). Enteroids (8 wells/animal) were maintained in proliferation media (CMGF+) for 4 d to promote the development of undifferentiated enteroid structures. On d 4, media without Wnt3a (CMFG‐ media) were added to induce differentiation and enteroids were cultured for 4 additional days when clear budding and crypt like structures were observed.

### 2.4 Citrulline production

In vivo citrulline production was calculated by non‐compartmental analysis using the [^15^N] citrulline tracer as previously described (Mohammad et al., 2020). In brief, a biexponential curve was fitted to the enrichment data and the area under the curve calculated. By dividing the tracer dose by this area under the curve, citrulline rate of appearance (production) was estimated. Enteroid citrulline production was determined by measuring the citrulline accumulated over 24 hr in the wells and normalizing for DNA content. The DNA content per well was assessed with Hoechst 33,258 dye and measured by fluorescence spectrophotometry (excitation: 350 nm, emission: 473 nm). Isotopic enrichments and concentrations of citrulline were determined by HESI‐LC‐MS/MS as previously described (Marini, 2011).

### 2.5 Gene expression

Proximal jejunum was homogenized in guanidinium thiocyanate‐phenol‐chloroform extraction solution (TRIzol, Life Technologies, Gaithersburg, MD) and RNA was isolated using Qiagen RNeasy Mini elute columns (Qiagen Chatsworth, CA). Enteroids RNA was extracted from two wells using a commercial kit (MicroElute Total RNA kit; Omega bio‐tek).

After cDNA synthesis, PCR amplification and gene differentiation expression were performed with SYBR Green1 on a multicolor Real‐Time PCR detection system (CFX96, BioRad Laboratories, Hercules CA). The expression of the following groups of genes was determined: 1. Genes that code for enzymes not related to the metabolism of arginine and citrulline (developmentally regulated intestinal genes): sucrase‐isomaltate (SI), lactase phlorizin hydrolase (LPH), and mucine 2 (MUC2); 2. Genes that code for enzymes involved in the synthesis of citrulline: N‐acetylglutamate synthase (NAGS), ornithine aminotransferase (OAT), carbamoylphosphate synthetase I (CPS1), and ornithine transcarbamylase (OTC); 3. Genes that code for enzymes involved in the use of citrulline for the synthesis of arginine: argininosuccinate synthase (ASS1) and argininosuccinate lyase (ASL); 4. Genes that code for enzymes involved in the utilization of arginine: arginase 1 (ARG1), arginase 2 (ARG2), and inducible nitric oxide synthase (iNOS). GAPDH expression was used to normalize the expression of the genes of interest. Primer sequences were previously validated and published elsewhere (Robinson et al., 2018). Results from jejunum and enteroids are expressed as fold change compared to either jejunum from term pigs at birth (0d) or enteroids derived from this age group, respectively, using the ΔΔCt method and primer efficiency (Fleige et al., 2006).

### 2.6 Statistics

Data were analyzed using the proc mixed statement of SAS with age (−10, −5, 0, 7, 35 d) and sex (male and female), and their interaction as fixed effects and litter as the random effect of the model. Because for the gene expression data, the variance were not always homogenous (Levene test) across the different age groups, the mixed model was conducted on the ranked variables (Kruskal‐Wallis test). Post‐hoc pairwise comparisons were conducted using Tukey's procedure. In addition, linear and quadratic pre‐planned orthogonal contrasts were used to assess the shape of the response of the different variables to age. Because of the uneven spacing of “age”, contrast coefficients were obtained using the ORPOL function of proc IML in SAS. The glmpower procedure of SAS was used to determine that a sample size of 6–8 pigs per treatment age had a statistical power > 0.80 to detect a 20% difference among means, assuming a standard deviation of ~ 0.15. Means ± SE or medians (interquartiles) are shown and type I error was set at 0.05.

## 3 RESULTS

**Figure 2:**
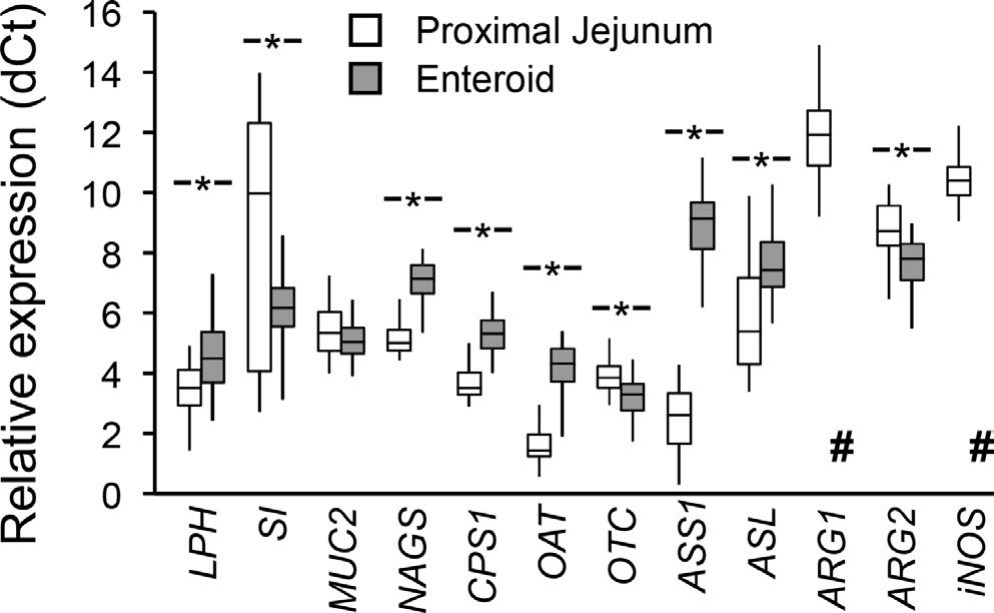
Relative gene expression (dCt; with respect to the housekeeping gene *GADPH*) of the genes of interest in proximal jejunum tissue and enteroids. Box‐plot and whiskers representation. Non‐parametric analysis (including age and sex as covariates) showed differences (marked with an asterisk, *p* < .001) between proximal jejunum and enteroids, with the exception of *MUC2* (*p* < .08). *n* = 38 pigs or enteroids; #, considered undetectable (Ct > 35)

The relative gene expression (dCt with respect to the housekeeping gene *GADPH*) of most of the target genes for the tissue aggregated data showed differences between proximal jejunum and enteroids (Figure 2); the exception was *MUC2* which was not different (*p* = .08). Gene expression was lower in enteroids (greater dCt; *p* < .001) for *LPH*, *ASS1*, *ASL*, *OAT*, *NAGS*, *CPS1* and expression for *ARG1* and *iNOS* was considered undetectable (Ct > 35). In contrast, gene expression of *SI*, *OTC*, *ARG2* was lower (*p* < .001) in proximal jejunum tissue than in enteroids.

**Figure 3:**
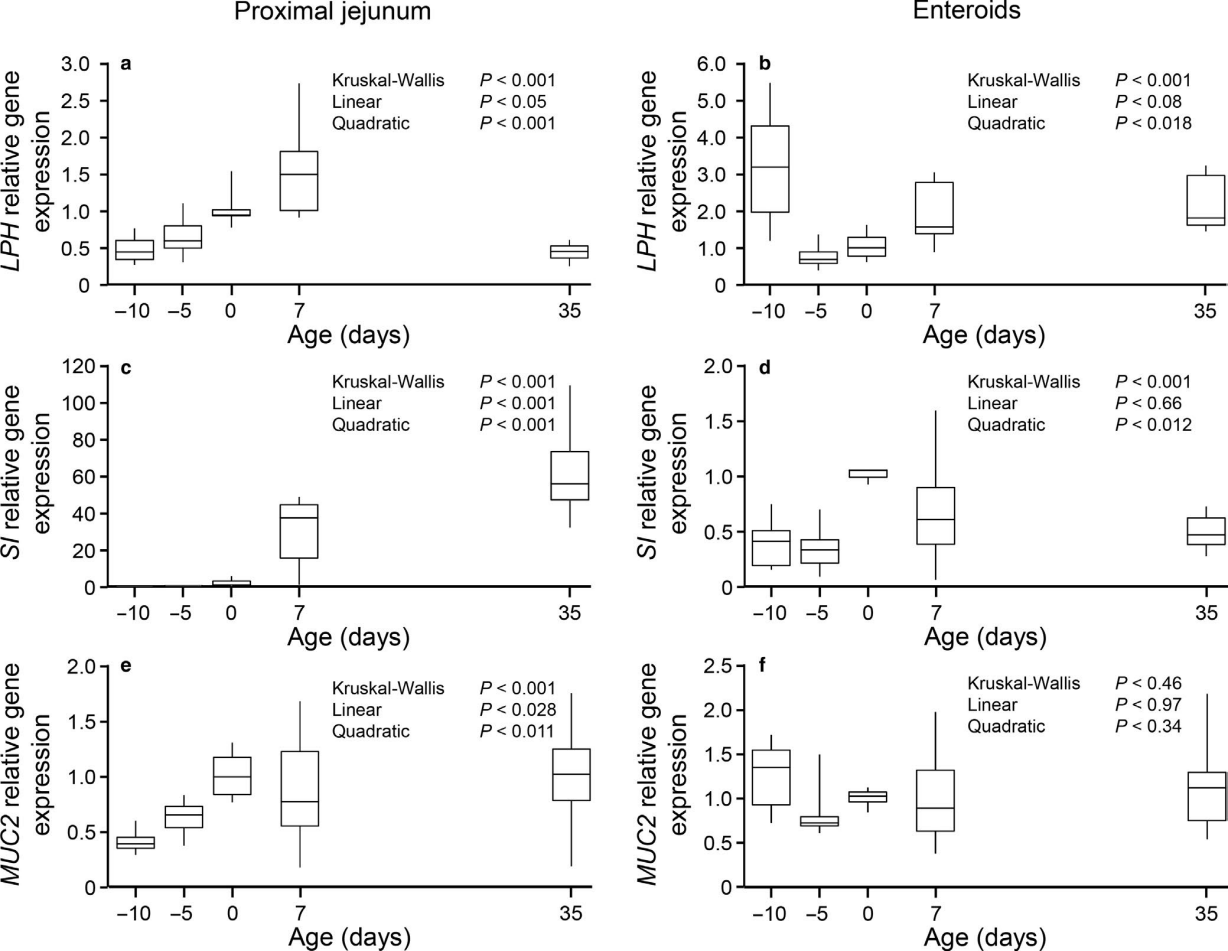
Gene expression of *LPH* (a and b), *SI* (c and d), and *MUC2* (e and f) in proximal jejunum tissue (a, c and e) and enteroids derived (b, d, and f) from pigs at different ages. Gene expression (fold, 2^‐ddCt^) was normalized using term pigs at birth (0day) as reference. Box‐plot and whiskers representation. *n* = 6–8 pigs. *LPH*, lactase phlorizin hydrolase; *SI*, sucrase isomaltase; *MUC2*, mucin 2

There was no sex effect for any of the variables analyzed and sex was removed from the statistical model. There was an age effect on the gene expression of *LPH* in proximal jejunum (*p* < .001). *LPH* expression increased to a maximum value at 7 d of age and then became minimal at 35 d (Figure 3a). In contrast, *SI* gene expression was undetectable in the gut of premature pigs at birth; *SI* expression then increased from 0 d reaching its maximum level at 35 d of age (*p* < .001 Figure 3c). *MUC2* showed a different expression pattern increasing linearly with age (*p* < .028) and reaching a plateau at 0 d (Figure 3e). The expression of *LPH*, *SI,* and *MUC2* followed a different pattern in enteroids. *LPH* gene expression showed a quadratic effect (*p* < .018) with a minimal expression in 5 d preterm and term pigs at birth; in contrast, *SI* expression also showed a quadratic effect of age (*p* < .012) but the maximal expression was in term pigs at birth (0 d). There was no effect of age (*p* = .46) on *MUC2* gene expression.

**Figure 4:**
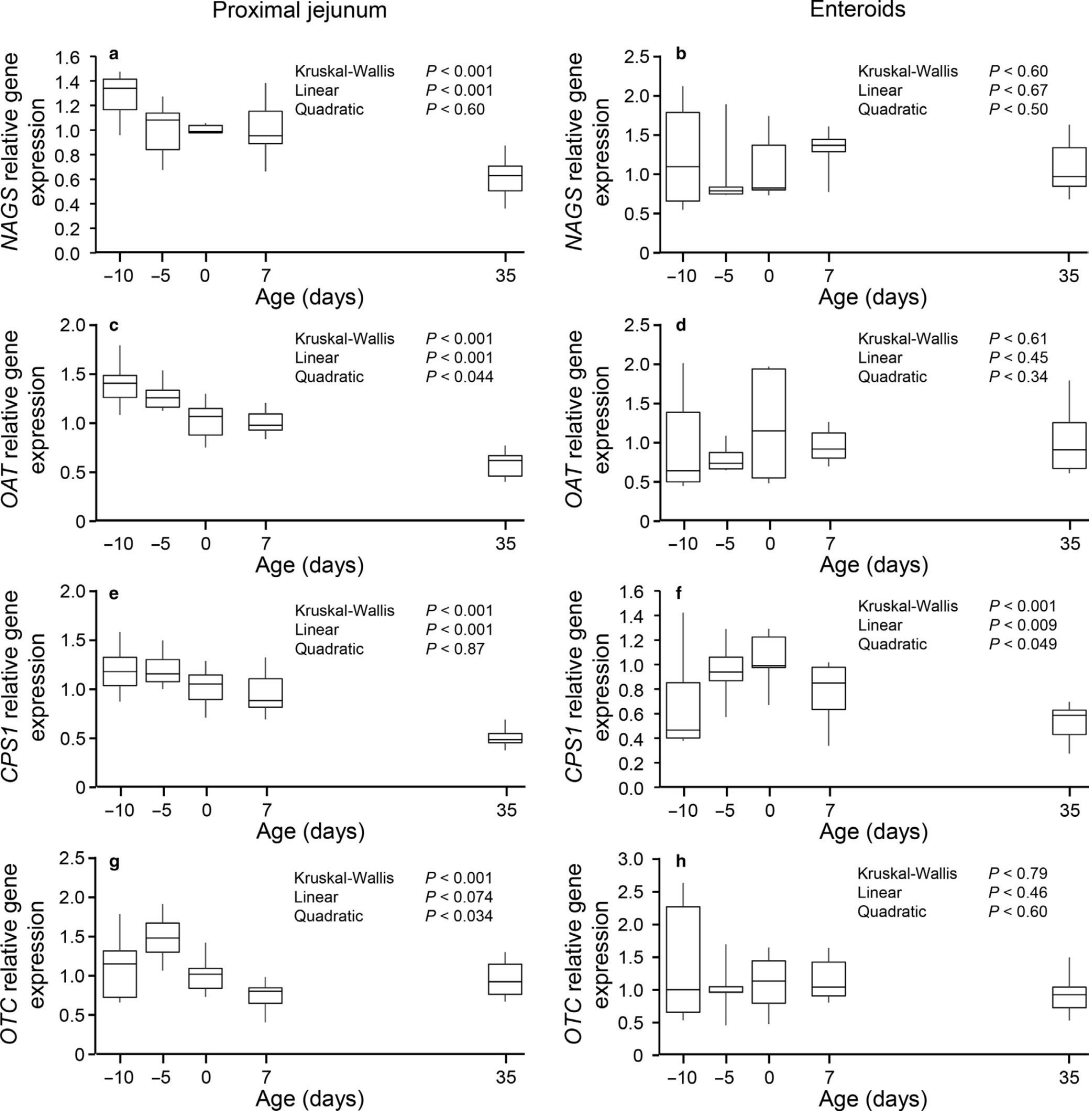
Gene expression of enzymes involved in the synthesis of citrulline (*NAGS*, a and b; *OAT*, c and d; *CPS1*, e and f; *OTC*, g and h) in proximal jejunum tissue (a, c, e, and g) and enteroids derived (b, d, f, and h) from pigs at different ages. Gene expression (fold, 2^‐ddCt^) was normalized using term pigs (0 day) as reference. Box‐plot and whiskers representation. *n* = 6–8 pigs. *NAGS*, N‐acetylglutamate synthase; *OAT*, ornithine aminotransferase; *CPS1,* carbamoyl phosphate synthetase 1; *OTC,* ornithine transcarbamylase

An age effect was observed for the proximal jejunum expression of genes coding for enzymes involved in the synthesis of citrulline (*p* < .001; Figure 4). This effect was linear for *NAGS*, *OAT,* and *CPS1* (*p* < .001) and showed a decrease in expression with age. For *OTC* expression a quadratic effect was shown (*p* < .034) with the highest expression at −5 d. In contrast, no age effect was detected (*p* > .60) on gene expression in enteroids, except for CPS1 which showed a linear (*p* < .009) and quadratic (*p* < .049) age effect with the maximal value observed at 0 d (term pigs at birth).

**Figure 5:**
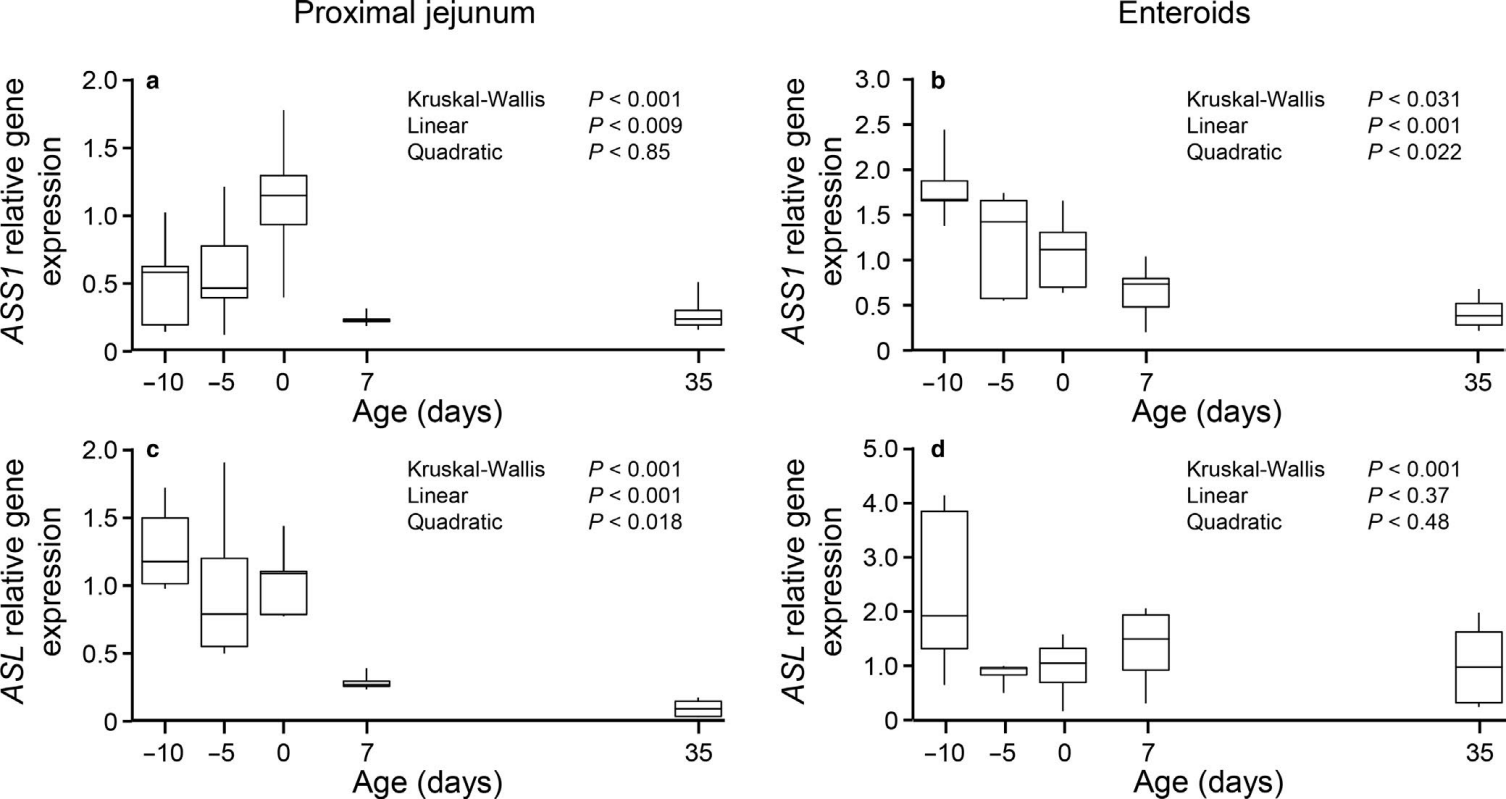
Gene expression of enzymes involved in the utilization of citrulline and the synthesis of arginine (*ASS1*, a and b; *ASL*, c and d) in proximal jejunum tissue (a and c) and enteroids derived (b and d) from pigs at different ages. Gene expression (fold, 2^‐ddCt^) was normalized using term pigs (0 day) as reference. Box‐plot and whiskers representation. *n* = 6–8 pigs. *ASS1*, argininosuccinate synthetase; *ASL*, argininosuccinate lyase

The proximal jejunum expression of genes coding for enzymes involved in the utilization of citrulline and the synthesis of arginine showed an age effect (*p* < .001; Figure 5). The effect was linear (*p* < .009) for both *ASS1* and *ASL* expression showing the lowest values at 7 and 35 d of age. Although enteroid *ASS1* expression decrease linearly (*p* < .05) and quadratically (*p* < .001) with age, no age effect (P + 0.46) was detected for *ASL* expression.

**Figure 6:**
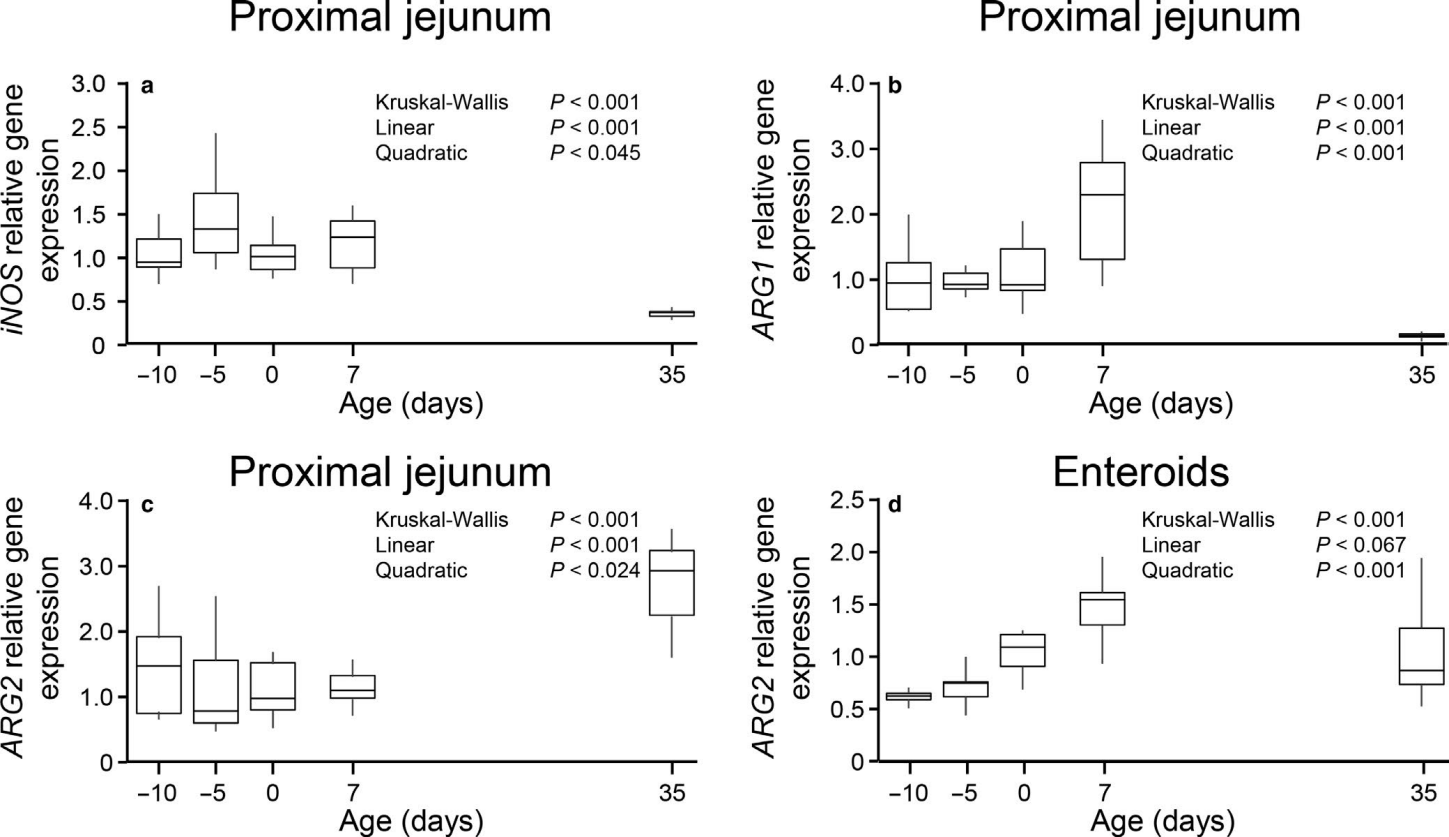
Gene expression of enzymes involved in the utilization of arginine (*iNOS*, a; *ARG1*, b; and *ARG2*, c and d) in proximal jejunum tissue (a, b, and c) and enteroids derived (d) from pigs at different ages. Gene expression (fold, 2^‐ddCt^) was normalized using term pigs (0 day) as reference. Box‐plot and whiskers representation. *n* = 6–8 pigs. *iNOS*, inducible nitric oxide synthase; *ARG1*, arginase 1; *ARG2*, arginase 2

An age effect was also observed for the proximal jejunum expression of genes coding for enzymes involved in the utilization of arginine (*p* < .001; Figure 6). Whereas linear (*p* < .001) and quadratic (0.001 < *p* < .045) effects were observed for *ARG1*, *ARG2* and *iNOS*, the gene expression was minimal for *ARG1* and *iNOS*, and maximal for *ARG2* at 35 d of age. The expression of *ARG1* and *iNOS* in enteroids was below the set cutoff quantitation limit (Ct > 35). A quadratic age effect (*p* < .001) was detected for the expression of *ARG2*, which was maximal at age 7 d (Figure 6d).

**Figure 7:**
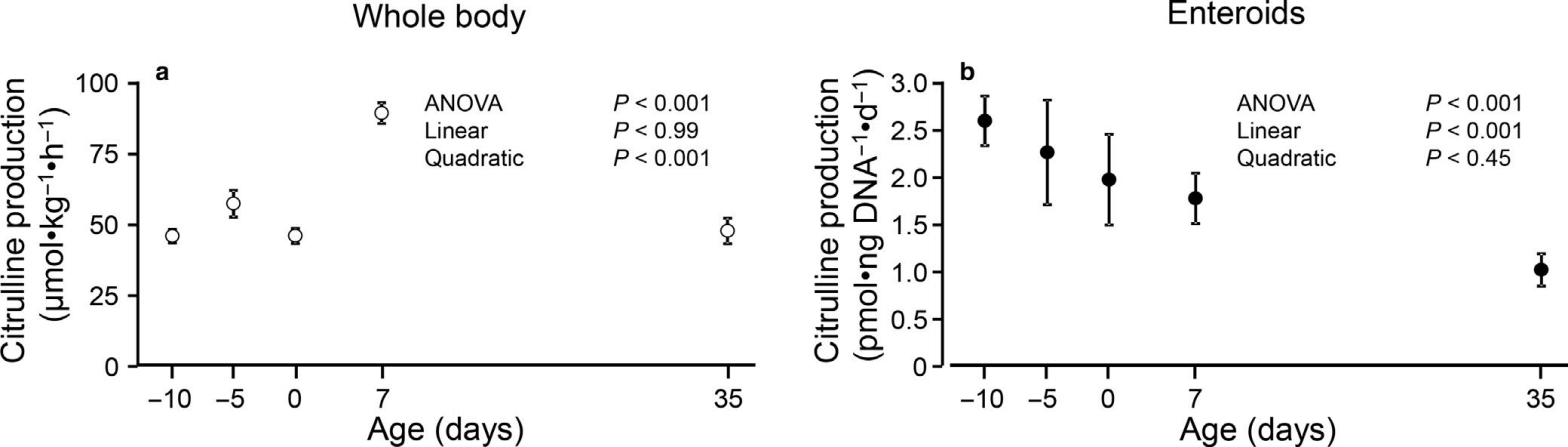
Whole body citrulline production in pigs at different ages (a) and by enteroids (b) derived from these pigs. Symbols represent means ± SE, *n* = 6–8 pigs. Citrulline production by enteroids was determined in triplicate and normalized using DNA content

Whole body citrulline production showed an age effect (*p* < .001), which was due to greater production at age 7 d (80 ± 3.3 µmol·kg^−1^·h^−1^) whereas it was similar at other ages (41–51 µmol·kg^−1^·h^−1^; Figure 7a). In contrast, an age‐dependent linear effect (*p* < .001) was found for citrulline production by enteroids, with a decrease in production as the age of the pigs increased (Figure 7b).

## 4 DISCUSSION

The developmental changes reported for the small intestine in the literature are, for the most part, confined to the single cell epithelial layer that lines this organ. These epithelial cells have marked qualitative differences along the crypt‐villus axis; stem cells proliferate in a crypt niche (containing also Paneth cells) and differentiate into enterocytes, goblet, and neuro‐endocrine cells which migrate lining the villus region (Chin, Hill, Aurora, & Spence, 2017). Despite the inclusion of these cells with multiple other cell types from other layers (e.g., muscular, serosal), gene expression profiles measured in whole intestinal tissue extracts are representative of epithelial cell‐only gene expression (Anderle et al., 2005). However, the presence of cells from other layers may result in the detection of certain genes in whole tissue which may be absent in the epithelial layer. For example, we found that *ARG1* was present in whole jejunal tissue but absent in enteroids. This is consistent with the lack of *ARG1* expression in epithelial cells (Jonge et al., 2002; Kohler et al., 2008) but with its expression in endothelial cells and macrophages.

An additional quantitative cephalocaudal axis on expression has been reported for many genes, including LPH, sucrase and enzymes involved in the synthesis of citrulline (Fan, Stoll, Jiang, & Burrin, 2001; Hurwitz & Kretchmer, 1986; Shulman, Henning, & Nichols, 1988); all of these genes have been shown to have a higher expression in proximal jejunum tapering toward the distal ileum. For these reasons, we collected proximal jejunum to determine gene expression and as the source for crypts in the establishment of enteroids.

As expected, we observed in jejunal tissue, an increase in *LPH* expression with age (with a subsequent decline after weaning) and in postnatal *SI* gene expression which achieved its greatest level after weaning. The expression pattern of *LPH* and *SI* have been well characterized in many species, including the pig (Sangild, Sjöström, Noren, Fowden, & Silver, 1995), and have been used as markers of gut maturation and development. Because *LPH* and *SI* are expressed in the villus region, and *MUC2* is expressed in mature goblet cells, the presence of transcripts for these three enzymes can also be used as a marker for cell differentiation in enteroids (Gonzalez, Williamson, Piedrahita, Blikslager, & Magness, 2013). Although *LPH* and *MUC2* were expressed at similar levels in whole tissue and enteroids, the enteroid expression failed to recapitulate the age‐dependent expression pattern observed in jejunum. Furthermore, enteroids from post‐weaned pigs failed to show the large increase in *SI* expression, which is characteristic of this developmental period.

Similarly, the age effect on the expression of the enzymes involved in the synthesis of citrulline was not present in enteroids whereas in whole tissue we were able to replicate patterns reported by other researchers (De Jonge et al., 1998; Herzfeld & Raper, 1976; Hurwitz & Kretchmer, 1986; Kohler et al., 2008; Wu, Knabe, et al., 2004). Despite the age‐dependent decrease in *NAGS*, *OAT* and *CPS1* expression in jejunum, whole body citrulline production remained fairly constant with the exception of 7‐d‐old pigs. This is consistent with our previous reports which showed a greater citrulline production in pigs of similar age (Marini et al., 2017; Marini, Stoll, Didelija, & Burrin, 2012). In contrast, we did not find differences in citrulline production observed previously between premature and term piglets (Robinson et al., 2018); however, Robinson et al. measured citrulline production in pigs receiving total parenteral nutrition and enteral feeding 48–72 hr after C‐section whereas in the present report pigs were studied immediately after birth (and before feeding). In enteroids, citrulline production showed a linear decrease with age which did not reflect the in vivo observations.

During the neonatal period the enzymes that utilize citrulline for the synthesis of arginine are present in the epithelial cells of the small intestine, but they are lost as the animal or infant matures (De Jonge et al., 1998; Herzfeld & Raper, 1976; Hurwitz & Kretchmer, 1986; Kohler et al., 2008; Wu, Knabe, et al., 2004). Whereas the tissue expression of *ASS1* and *ASL* followed this developmental pattern reported by others, enteroids failed to recapitulate these changes. The *ASS1* expression was lower in enteroids derived from older pigs, although the expression in enteroids from preterm and term pigs at birth showed a decrease with gestational age whereas the opposite was observed in the jejunum.

As expected, the gene expression for some of the enzymes that utilize arginine (*ARG1* and *iNOS*) was negligible in enteroids, but present in jejunal tissue. Although, *iNOS* can be expressed by epithelial cells when challenged with endotoxin, under basal conditions its expression is minimal (Morin, Unno, Hodin, & Fink, 1998); *ARG1*, in contrast, is not expressed by epithelial cells (Jonge et al., 2002). The tissue expression of *ARG2* was lower in the neonatal and suckling pigs but increased in post‐weaning animals, consistent with reports by others (Herzfeld & Raper, 1976; Hurwitz & Kretchmer, 1986). In contrast, *ARG2* expression by enteroids increased with age reaching a maximum value in 7‐d‐old animals.

The developmental changes in the gastrointestinal tract result from the interaction of four major determinants: genetic endowment, intrinsic developmental, and biologic clock, endogenous regulatory mechanisms and environmental influences (Lebenthal 1999). Although the intrinsic programming of crypt stem cells is sufficient to replicate the molecular and physiological profiles of the segment from where they originated (“location memory”) (Kozuka et al., 2017; Middendorp et al., 2014), it seems that additional external cues are needed to recreate the age‐dependent phenotype. The timing of these cues may be also important. For example, crypt cells obtained from older pigs seemed to be more Wnt‐dependent than those collected from neonatal animals (Khalil et al., 2016). However, this may be needed only initially during the establishment of the culture since we showed here that removing Wnt3a after 4 days did not affect viability and induced differentiation. Regardless, novel developments in the culture of enteroids, such as co‐culture with subepithelial myofibroblasts (Khalil et al., 2016), culture in two‐dimensional monolayer (Kozuka et al., 2017), genetic modifications (Khalil et al., 2016; Wang, Yuan, Didelija, Mohammad, & Marini, 2018), inclusion of microbiota (Chen, Zhou, Roh, Estes, & Kaplan, 2017), and manipulation of the culture media may be needed to recreate age‐dependent gene expression.

In conclusion, 3D enteroids derived from jejunal crypts of pigs at different ages and cultured under the set of conditions described above failed to replicate the gene expression observed in whole tissue and whole body citrulline production. Additional external cues may be needed to reproduce the age‐dependent phenotype. Alternatively, these external cues may be sufficient to recreate the gene expression seen at different ages.

## CONFLICT OF INTEREST

No conflicts of interest, finance, or otherwise, are declared by the authors.

## AUTHOR CONTRIBUTIONS

DGB and JCM designed research. JCM wrote the manuscript and has primary responsibility for final content. MAM, ICD, BS, and JCM conducted animal research and analyzed samples. JCM analyzed data and performed statistical analysis. All authors have read and approved the final manuscript.
